# Child and neighborhood factors associated with pediatric injuries sustained while engaged in activities where helmet usage is recommended

**DOI:** 10.1186/s40621-025-00598-6

**Published:** 2025-07-07

**Authors:** Brent M. Troy, Maneesha Agarwal, Allison F. Linden, Andrew Jergel, Anthony Giarusso, Kiesha Fraser Doh

**Affiliations:** 1 Dell Medical School, Department of Pediatrics, The University of Texas at Austin, Austin, Texas 78717 USA; 2 Department of Pediatrics, Division of Emergency, Medicine/Children, Emory University School of Medicine, Healthcare of Atlanta, Atlanta, Georgia USA; 3 Department of Surgery, Division of Pediatric, Surgery/Children’s, Emory University School of Medicine, Healthcare of Atlanta, Atlanta, Georgia USA; 4 Pediatric Biostatistics Core, Childrens Healthcare of Atlanta Emory University, Atlanta, Georgia USA; 5 Georgia Institute of Technology, Atlanta, Georgia USA

**Keywords:** Helmet, Bicycle, ATV, Traumatic Brain Injury, GIS Mapping, Pediatrics

## Abstract

**Background:**

Unintentional injuries, including traumatic brain injuries (TBI) during activities where helmet usage is recommended (AWHUR), are a leading cause of pediatric morbidity and mortality in the U.S. While advocacy and education are proven measures to address safety, community resources in a child’s neighborhood are known to have a large impact on their health.

**Methods:**

We utilized the trauma registry at two pediatric trauma centers, in a major metropolitan area from 2018 to 2022, to perform a retrospective chart review and Geographical Information System (GIS) mapping on all AWHUR injuries that were included in the trauma registry. Data extracted from the trauma registry included: mechanism, demographics, insurance status, and injury characteristics. AWHUR data was then overlaid with the Childhood Opportunity Index (COI) to assess community resources in relation to injury characteristics.

**Results:**

Our sample size included 1425 children throughout the 5-year time period. The most common injury mechanisms included: bicycle 34.0%, ATV 18.2%, skateboard 13.3%, scooter 9.2%, and dirt-bike 7.4%. Most patients in very low and low COI were publicly insured, respectively 81.9% and 63.2%; while 65.8% of high COI injured patients were privately insured children. There was a statistically significant difference in helmet usage across different levels of COI (p < 0.001). The rates of helmet usage by COI ranking from very low to very high were as follows: 21.6%, 25.2%, 37.8%, 40.2%, and 51.6% utilization. Among those injured while wearing a helmet, the odds of sustaining a higher ISS were 34% lower (OR = 0.66, 95% CI: 0.50—0.89) compared to those who were not wearing a helmet at the time of injury. Additionally, GIS mapping identified low and very low COI communities with higher injury rates and lower helmet use.

**Conclusion:**

Children with lower COI were more likely to be publicly insured with a lower percentage of helmet usage. Overlapping injury data and COI identified high-risk communities where low resources can contribute to growing injury severity. This data can then be used to inform injury prevention and highlight the importance of community factors.

## Background

Unintentional injuries, including traumatic brain injuries (TBI) during activities where helmet usage is recommended (AWHUR), are a leading cause of pediatric morbidity and mortality in the United States [1]. Specifically, TBIs are a leading cause of death and disability amongst children and adolescents in the U.S. [2]. These injuries can commonly occur for children participating in recreational AWHURs including bicycling, scootering, riding all-terrain vehicles (ATVs), snow-sports (skiing, snowboarding), skating (ice, roller, blading), skateboarding, and participation in equestrian sports. Combined, these activities have resulted in over 42,000 pediatric ED visits annually for nonfatal TBI, with bicycle injuries causing the highest incidence of TBI [2, 3]. Concerningly though, there has been an increase in pediatric TBI-related emergency department (ED) visits over recent years, as well as an increase in concussions diagnosed in the ED [4]. In 2014, there were > 830,000 ED visits and hospitalizations, and > 2,500 deaths in children due to TBI [5, 6].

Analysis has also shown that the geographical location of the injury can be associated with the severity of injury and lead to potential delays in care or mis-triage at a non-trauma center [7]. Additionally, rural TBIs are at higher risk for dangerous injury mechanisms and trauma severity when compared to pediatric patients injured in an urban setting [7]. Rural patients were more likely to have unnecessary transfers leading to increased costs as well as inadequate initial evaluation in non-trauma rural EDs. The location of the injury has also been associated with mortality rates four times higher than expected in non-urban areas [8]. Analysis has also shown that there are significant disparities in access to pediatric trauma centers based on race and ethnicity that leave children at risk for poor trauma outcomes [9]. Through Geospatial Information Systems (GIS) mapping of trauma registries, there have been areas identified as high risk for injury [10].

While participation in the above-mentioned AWHUR activities is part of pediatric well-being, helmets have been proven to reduce the incident and severity of TBIs [11–15]. The Center for Disease Control and Prevention, as well as The American Academy of Pediatrics, have published policies on the importance of proper helmet usage during common activities of childhood [16]. We also know that interventions in communities have been shown to increase awareness and helmet usage for children [17–21]. However, these costly efforts should be focused on targeted problematic and high-risk areas [22, 23]. The Childhood Opportunity Index (COI) is a database measuring and mapping the quality of resources in a child’s neighborhood that are important for healthy development. Previous analysis has identified that pediatric patients presenting to the pediatric ED with lower COI had a higher proportion of mortality [24]. In addition, studies have determined that children from lower COI neighborhoods are more likely to be re-admitted to the pediatric intensive care unit after previous discharge, especially those with chronic medical conditions [25].

Thus, our primary aim was to utilize geographic markers to identify AWHUR injury characteristics and demographics in the communities of interest. We additionally aimed to compare census tracts where head injuries are more prevalent based on neighborhood resources utilizing the COI. We hypothesized that there are specific areas (hotspots) where there is a lower incidence of helmet usage amongst children ages 0 to 18 years old engaging in AWHUR within the metropolitan area. We additionally hypothesize that children, who presented after sustaining an injury while unhelmeted and engaging in AWHUR, are more likely to reside in areas with less resources thus having a lower COI score denoting the limitations of their neighborhood resources as defined by COI.

## Methods

### Study overview

We performed a cross-sectional retrospective chart review of the trauma registry at a Level 1 and at a Level 2 pediatric trauma center within a single pediatric healthcare system serving a major metropolitan area. These emergency departments combined care for approximately 180,000 patients annually. AWHUR injuries were identified through analysis of the trauma registry along with their associated demographics and injury characteristics. AWHUR were defined as activities involving bicycling, scootering (powered and un-powered, including hoverboards), off-road-vehicles (ATVs, go-karts, etc.), skiing, snowboarding, ice skating, roller skating, rollerblading, skateboarding, and equestrian sports. This data was then analyzed employing the COI 2.0 in conjunction with Geographical Information System (GIS) mapping. This study received approval via the Children’s Healthcare of Atlanta institutional review board (IRB).

### Study population

Children aged 0 to 18 years old who presented or were transferred to the Level 1 and Level 2 trauma center emergency departments from 2018–2022, and were included in the trauma registry, were eligible for the study. The trauma registry inclusion criteria are defined by the American College of Surgeons National Trauma Standard Data Set. The main patient qualifications to be included in the registry are: one or more traumatic injuries sustained within the previous 14 days of a hospital encounter; direct hospital admission or transferred from another facility via emergency medical services (EMS) or air ambulance; death in the ED; and presentation as a trauma activation requiring surgical presence per hospital guidelines.

Patients from the trauma registry were included in our evaluation based on the following International Classification of Diseases (ICD)−10 codes: S00-S99, T07, T14, and T79.A1-T79.A9 with one of the injuries in the following codes: S00, S10, S20, S30, S40, S50, S60, S70, S80, and S90 (Table 1). Additionally in the initial evaluation, V00-V99 ICD-10 coded patients were included based on their external mechanism while S00-S99 provided us patients based on their presenting injury. Initial patient eligibility was confirmed via brief patient injury summaries found in the trauma registry, and patients queried from the registry via ICD-10 codes without a clear injury mechanism from the trauma registry summary were individually chart reviewed to determine if a patient’s presentation was consistent with an AWHUR.

**Table 1:** Activities where helmet use is recommended ICD-10 codes

ICD 10 Codes	
T07	Inuires involving multiple body regions
T14	Injury of unspecificed body region
T79	Certain early complications of trauma
S00-S09	Injuries to the head
S10-S19	Injuries to the neck
S20-S29	Injuries to the thorax
S30-S39	Injuries to the abdomen, lower back, lumbar spine, pelvis, and external genitals
S40-S49	Injuries to the shoulder and upper arm
S50-S59	Injuries to the elbow and forearm
S60-S69	Injuries to the wrist, hand, and fingers
S70-S79	Injuries to the hip and thigh
S80-S89	Injuires to the knee and lower leg
S90-S99	Injuires to the ankle and foot
V00-V09	Pedestrian injured in transport accident
V10-V19	Pedal cycle rider injuried in transport accident
V20-V29	Motorcycle rider injuried in transport accident
V30-V39	Occupant of three-wheeled motor vehicle injured in transport accident
V60-V69	Occupant of heavy transport vehicle injured in transport accident
V80-V89	Other land transport accidents
V98-V99	Other and unspecified transport accidents

This table lists the ICD-10 codes that were used to obtain patients from the trauma registry

Data abstracted from the trauma registry included demographics such as race, ethnicity, gender, age, insurance status, and home address, as well as injury characteristics including injury severity score (ISS), activity type, helmet use, presence of head injury, neurosurgical consultation, and extent of head injury. Head injuries were identified through the Glasgow Coma Scale (GCS) and ICD codes consistent with a head injury. Race and ethnicity were reported by the caregiver typically. If a child presented with unknown helmet status, and helmet status could not be confirmed via chart review, these patients were included in mechanism analysis, but they were excluded from further injury characteristics such as head injury and helmet status.

Exclusion criteria for this study included patients who were older than 18 years of age, or patients who presented via an injury mechanism not included in above-mentioned AWHUR criteria. Children who were not directly participating in the AWHUR were excluded (e.g., a patient struck by another child riding a bicycle). Additionally, children with addresses not in Georgia, as well as those with incomplete addresses, were also removed from the database for standardization of the analysis.

### Child opportunity index

The COI 2.0 is a measurement of the resources a child has access to in their neighborhood [26]. The COI gives a ranking from highest to lowest for access to resources and healthy opportunities, such as education, healthcare, green space, nutritious food, and socio-economic factors, in the child’s neighborhood. The COI has an overall composite score based on 29 community characteristics that comprise the ranking for each neighborhood, and it is then categorized into 3 sub-domains: education, health and/or environment, and social and/or economic.

Census tract level analysis was incorporated to compare our patient home addresses and zip codes with the COI. The COI has 5 main categorizations of the resources in a community ranging from very low to very high. A very low COI indicated that a child’s zip code level data places them in the highest community-level vulnerability. Our study compared patient demographics (i.e. race, gender, and insurance status) and injury characteristics in conjunction with the patient’s COI score.

### Data analysis

Descriptive Statistics were summarized for categorical data using counts and percentages. Group comparisons between the different levels of COI, years, and helmet usage utilized Pearson’s Chi-squared test and Fisher’s exact test (when cell counts were less than 5). We quantified the association between the primary outcomes—Injury Severity Score (ISS) and helmet use—and the covariates mechanism of injury and activity type using univariate and multivariable logistic regression models. All multivariable analyses were then adjusted for potential confounders (determined using a priori methods), including race and insurance status. odd ratios (OR) and 95% Confidence Intervals (CI) were provided. All p-values less than 0.05 were considered statistically significant. All data cleaning and statistical testing was performed in R Statistical Software (v4.2.1; R Core Team 2022).

### Geographic Information Systems (GIS) mapping

Two trauma centers in metropolitan Atlanta provided home addresses of 1,425 pediatric head trauma patients between 2018–2022. ArcGIS Pro 2.1 was used to geocode these addresses. In addition to home addresses, the injury mechanism, gender, race, ethnicity, insurance status, helmet usage, and other injury characteristics were also included in the database for analytic purposes. Once geocoded, patient locations were spatially joined to county boundaries within the 22-county Atlanta Metropolitan Statistical Area (MSA) to create various aggregate county-level maps, such as injury rates by helmet usage, by injury mechanism, or by visit date. The geocoded addresses were also spatially joined or aggregated to the Child Opportunity Index data layer boundaries, a census tract-level composite index of children’s neighborhood opportunity that contains 44 indicators related to education, health, environment, and socioeconomics. This allowed for a direct comparison between the COI index and the aggregated counts of head trauma patients and their trauma registry specific variables at the census tract level.

## Results

### AWHUR demographics

Over a 5-year period, 1,425 patients were analyzed from the trauma registry. 288 patients in 2018 up to 347 in 2019, 510 in 2020, 365 in 2021 and 314 in 2022 (Table 2). Over half (68.8%) of the patients who presented more than 5 years after AWHUR were male. One-third of the patients were between 5 and 9 years old, and 49% were between 10 and 14 years of age over the course of the 5 years. Throughout the 5 years, 50.8% of patients were publicly insured, while 40.9% were children with private or commercial insurance. The patient population throughout the study was inclusive, with 62.9% identifying as White, 27% identifying as Black, and 4% as Asian.

**Table 2:** Activities where helmet use is recommended patient characteristics patient demographics

		Overall, n = 1425	2018, n = 239	2019, n = 274	2020, n = 407	2021, n = 273	2022, n = 232	p-value
Age (%)	< = 4 yrs	112 (7.8%)	19 (7.9%)	23 (8.4%)	35 (8.6%)	16 (5.9%)	19 (8.2%)	0.009
	5–9 yrs	460 (32.2%)	85 (35.6%)	89 (32.4%)	135 (33.2%)	80 (29.3%)	71 (30.6%)	
	10–14 yrs	694 (48.7%)	120 (50.2%)	144 (52.6%)	192 (47.1%)	132 (48.3%)	106 (45.7%)	
	15–18 yrs	159 (11.3%)	15 (6.3%)	18 (6.6%)	45 (11.1%)	45 (16.5%)	36 (15.5%)	
Gender (%)	Female	449 (31.5%)	87 (36.4%)	84 (30.7%)	142 (34.9%)	80 (29.3%)	56 (24.1%)	0.023
	Male	976 (68.5%)	152 (63.6%)	190 (69.3%)	265 (65.1%)	193 (70.7%)	176 (75.9%)	
Insurance (%)	Public	724 (50.8%)	92 (38.5%)	130 (47.4%)	189 (46.4%)	156 (57.1%)	157 (67.7%)	< 0.001
	Private/commerical insurance	583 (40.9%)	111 (46.4%)	117 (42.7%)	174 (42.8%)	110 (40.3%)	71 (30.6%)	
	Other	118 (8.3%)	36 (15.1%)	27 (9.9%)	44 (10.8%)	7 (2.6%)	4 (1.7%)	
Race (%)	White	883 (62.9%)	158 (66.1%)	173 (63.1%)	255 (63.9%)	154 (58.1%)	143 (63.0%)	0.787
	Black or African American	379 (27.0%)	62 (25.9%)	72 (26.4%)	100 (25.1%)	82 (30.9%)	63 (27.8%)	
	Asian	56 (4.0%)	6 (2.5%)	10 (3.6%)	21 (5.3%)	12 (4.5%)	7 (3.1%)	
	Other Race	86 (6.1%)	13 (5.5%)	19 (6.9%)	23 (5.7%)	17 (6.5%)	14 (6.1%)	
Ethnicity (%)	Hispanic or Latino	173 (12.2%)	28 (11.7%)	40 (14.6%)	50 (12.3%)	31 (11.4%)	24 (10.3%)	0.655
	Not Hispanic or Latino	1250 (87.8%)	211 (88.3%)	234 (85.4%)	356 (87.7%)	241 (88.6%)	208 (89.7%)	
Mechanism (%)	ATV	260 (18.2%)	26 (10.9%)	49 (17.9%)	70 (17.2%)	57 (20.9%)	58 (25.0%)	< 0.001
	Bicycle	484 (34.0%)	88 (36.8%)	100 (36.5%)	164 (40.3%)	78 (28.6%)	54 (23.3%)	
	Dirt bike	106 (7.4%)	17 (7.1%)	15 (5.5%)	23 (5.7%)	26 (9.5%)	25 (10.8%)	
	Electric scooter	25 (1.7%)	4 (1.7%)	5 (1.8%)	5 (1.2%)	7 (2.6%)	4 (1.7%)	
	Go cart	29 (2.0%)	5 (2.1%)	11 (4.0%)	1 (0.2%)	5 (1.8%)	7 (3.0%)	
	Horse	48 (3.4%)	18 (7.5%)	8 (2.9%)	7 (1.7%)	3 (1.1%)	12 (5.2%)	
	Hoverboard	62 (4.4%)	10 (4.2%)	10 (3.6%)	16 (3.9%)	16 (5.9%)	10 (4.3%)	
	Other	35 (2.5%)	10 (4.2%)	3 (1.1%)	6 (1.5%)	9 (3.3%)	7 (3.0%)	
	Roller skating	37 (2.6%)	7 (2.9%)	12 (4.4%)	7 (1.7%)	4 (1.5%)	7 (3.0%)	
	Rollerblading	18 (1.3%)	4 (1.7%)	7 (2.6%)	3 (0.7%)	2 (0.7%)	2 (0.9%)	
	Scooter	131 (9.2%)	22 (9.2%)	21 (7.7%)	45 (11.2%)	21 (7.7%)	22 (9.5%)	
	Skateboard	189 (13.2%)	28 (11.7%)	32 (11.7%)	60 (14.7%)	45 (16.4%)	24 (10.3%)	
	Snowboarding	1 (0.1%)	0 (0.0%)	1 (0.3%)	0 (0.0%)	0 (0.0%)	0 (0.0%)	
Helmet usage (%)	Helmet	404 (38.7%)	65 (39.6%)	73 (38.0%)	126 (38.7%)	72 (36.7%)	68 (40.7%)	0.951
	No	641 (61.3%)	99 (60.4%)	119 (62.0%)	200 (61.3%)	124 (63.3%)	99 (59.3%)	

AWHUR table examining patient characteristics over the years of the trauma registry

### Childhood opportunity index demographics

When examining the COI demographics for our study, 381 (26.7%) children resided in low or very low COI neighborhoods, compared to 784 (55%) children in high or very high COI neighborhoods (Table 3). The majority of black patients (78.7%) were in a very low COI, while the majority of white patients (77.7%) were in a very high COI. Most patients in very low and low COI were publicly insured, at 81.9% and 63.2%, respectively, while 65.8% of high COI injured patients were privately insured (p < 0.001).

**Table 3:** Activities where helmet use is recommended patient characteristics by childhood opportunity index

		Overall, n = 1425	Very Low, n = 210	Low, n = 171	Moderate, n = 260	High, n = 357	Very High, n = 427	p-value
Age (%)	< = 4 yrs	112 (7.8)	24 (11.4)	9 (5.2)	28 (10.8)	20 (5.6)	31 (7.3)	0.093
	5–9 yrs	460 (32.2)	77 (36.7)	59 (34.5)	71 (27.3)	115 (32.2)	138 (32.3)	
	10–14 yrs	694 (48.7)	92 (43.8)	88 (51.5)	129 (49.6)	177 (49.6)	208 (48.7)	
	15–18 yrs	159 (11.3)	17 (8.1)	15 (8.8)	32 (12.3)	45 (12.6)	50 (11.7)	
Gender (%)	Female	449 (31.5)	55 (26.2)	46 (26.9)	80 (30.8)	104 (29.1)	164 (38.4)	0.005
	Male	976 (68.5)	155 (73.8)	125 (73.1)	180 (69.2)	253 (70.9)	263 (61.6)	
Insurance (%)	Public	724 (50.8)	172 (81.9)	108 (63.2)	153 (58.8)	187 (52.4)	104 (24.4)	< 0.001
	Other	118 (8.3)	14 (6.7)	13 (7.6)	20 (7.7)	29 (8.1)	42 (9.8)	
	Private or Commerical	583 (40.9)	24 (11.4)	50 (29.2)	87 (33.5)	141 (39.5)	281 (65.8)	
Race (%)	White	883 (62.9)	32 (15.5)	103 (62.0)	170 (66.1)	251 (71.1)	327 (77.7)	
	Black or African American	379 (27.0)	163 (78.7)	50 (30.2)	72 (28.0)	60 (17.0)	34 (8.1)	
	Asian	56 (4.0)	2 (1.0)	2 (1.2)	4 (1.6)	10 (2.8)	38 (9.0)	
	Other Race	86 (6.1)	10 (4.8)	11 (6.6)	11 (4.3)	32 (9.1)	22 (5.2)	
Ethnicity (%)	Hispanic or Latino	173 (12.2)	20 (9.5)	30 (17.5)	20 (7.7)	63 (17.6)	40 (9.4)	< 0.001
	Not Hispanic or Latino	1250 (87.8)	190 (90.5)	141 (82.5)	240 (92.3)	294 (82.4)	385 (90.6)	
Helmet usage (%)	Helmet	404 (38.7)	32 (21.6)	32 (25.2)	76 (37.8)	105 (40.2)	159 (51.6)	< 0.001
	No	641 (61.3)	116 (78.4)	95 (74.8)	125 (62.2)	156 (59.8)	149 (48.4)	
Head injury (%)	Yes	415 (29.1)	64 (30.5)	60 (35.1)	77 (29.6)	101 (28.3)	113 (26.5)	0.314
	No	1010 (70.9)	146 (69.5)	111 (64.9)	183 (70.4)	256 (71.7)	314 (73.5)	
ISS categories (%)	Mild (1–8)	1017 (71.8)	156 (75.7)	116 (67.8)	184 (70.8)	240 (67.4)	321 (75.9)	
	Moderate (9–15)	309 (21.8)	37 (18.0)	42 (24.6)	56 (21.5)	91 (25.6)	83 (19.6)	
	Severe (16–24)	66 (4.7)	8 (3.9)	9 (5.3)	14 (5.4)	19 (5.3)	16 (3.8)	
	Very severe (25—greater)	24 (1.7)	5 (2.4)	4 (2.3)	6 (2.3)	6 (1.7)	3 (0.7)	
Mechanism (%)	ATV	260 (18.2)	34 (16.2)	50 (29.3)	70 (26.9)	63 (17.6)	43 (10.1)	
	Bicycle	484 (34.0)	85 (40.4)	57 (33.3)	68 (26.2)	115 (32.2)	159 (37.2)	
	Dirt bike	106 (7.4)	22 (10.5)	13 (7.6)	27 (10.4)	21 (5.9)	23 (5.4)	
	Electric scooter	25 (1.7)	5 (2.4)	1 (0.6)	5 (1.8)	8 (2.2)	6 (1.4)	
	Go cart	29 (2.0)	7 (3.3)	5 (2.9)	5 (1.8)	7 (2.0)	5 (1.2)	
	Horse	48 (3.4)	2 (1.0)	6 (3.5)	8 (3.1)	7 (2.0)	25 (5.9)	
	Hoverboard	62 (4.4)	11 (5.2)	10 (5.8)	8 (3.1)	12 (3.4)	21 (4.9)	
	Other	35 (2.5)	6 (2.9)	4 (2.3)	8 (3.1)	8 (2.2)	9 (2.1)	
	Roller skating	37 (2.6)	5 (2.4)	3 (1.8)	9 (3.5)	15 (4.2)	5 (1.2)	
	Rollerblading	18 (1.3)	4 (1.9)	0 (0.0)	3 (1.2)	6 (1.7)	5 (1.2)	
	Scooter	131 (9.2)	16 (7.6)	6 (3.5)	21 (8.1)	34 (9.5)	54 (12.6)	
	Skateboard	189 (13.2)	13 (6.2)	16 (9.4)	28 (10.8)	61 (17.1)	71 (16.6)	
	Snowboarding	1 (0.1)	0 (0.0)	0 (0.0)	0 (0.0)	0 (0.0)	1 (0.2)	

AWHUR table looking at patient characteristics in relation to their childhood opportunity index resources

### AWHUR injury characteristics

Over the 5-year time, the five most common injury mechanisms (bicycle, ATV, skateboard, scooter, and dirt-bike) accounted for 82.1% of all injuries. The most common injury mechanism was bicycle injuries at 34.0%, followed by ATV injuries at 18.2%, and skateboard related injuries at 13.3%. Additional injury severity characteristics did not find statistically significant results including neurosurgery consultation, Injury Severity Score, Glasgow Coma Scale, and head injury documentation.

### Childhood opportunity index injury characteristics

**Fig. 1:**
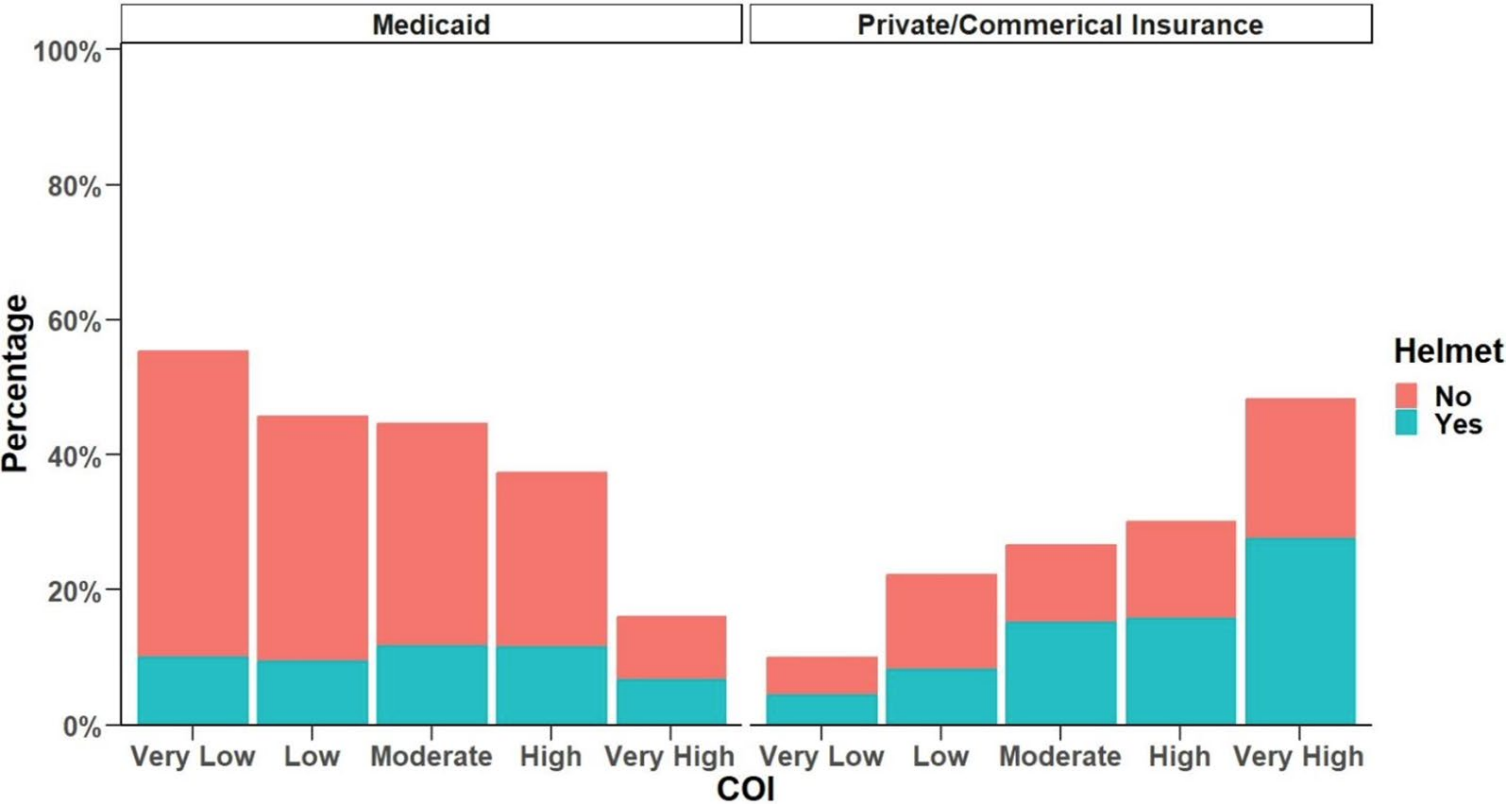
Helmet usage for each level of childhood opportunity index by insurance type (only medicaid & private/commercial). This graph demonstrates each COI resource category by percentage for public insurance and private/commercial insurance

There was a statistically significant difference in helmet usage across different levels of COI (p < 0.001). For AWHUR injury patients, those with very low COI had 21.6% helmet usage, low COI had 25.2% utilization, moderate COI had 37.8%, high COI had 40.2%, and very high COI had 51.6% utilization. (Table 3). In the unadjusted models, higher COI tended to show higher helmet usage. Children who lived in very high COI neighborhoods were 3.87 (95% CI 2.49—6.15) times more likely to utilize helmets compared to those with very low COI. However, after adjusting for race and insurance status, this association became insignificant with an OR of 1.33 (95% CI 0.77—2.32). Among those injured while wearing a helmet, the odds of sustaining a higher ISS were 34% lower (OR = 0.66, 95% CI: 0.50—0.89) compared to those who were not wearing a helmet at the time of injury. Figure 1 represents helmet usage in relation to COI for public and private insurance demonstrating the large gap in the parameters.

### Geographical information systems mapping

**Fig. 2:**
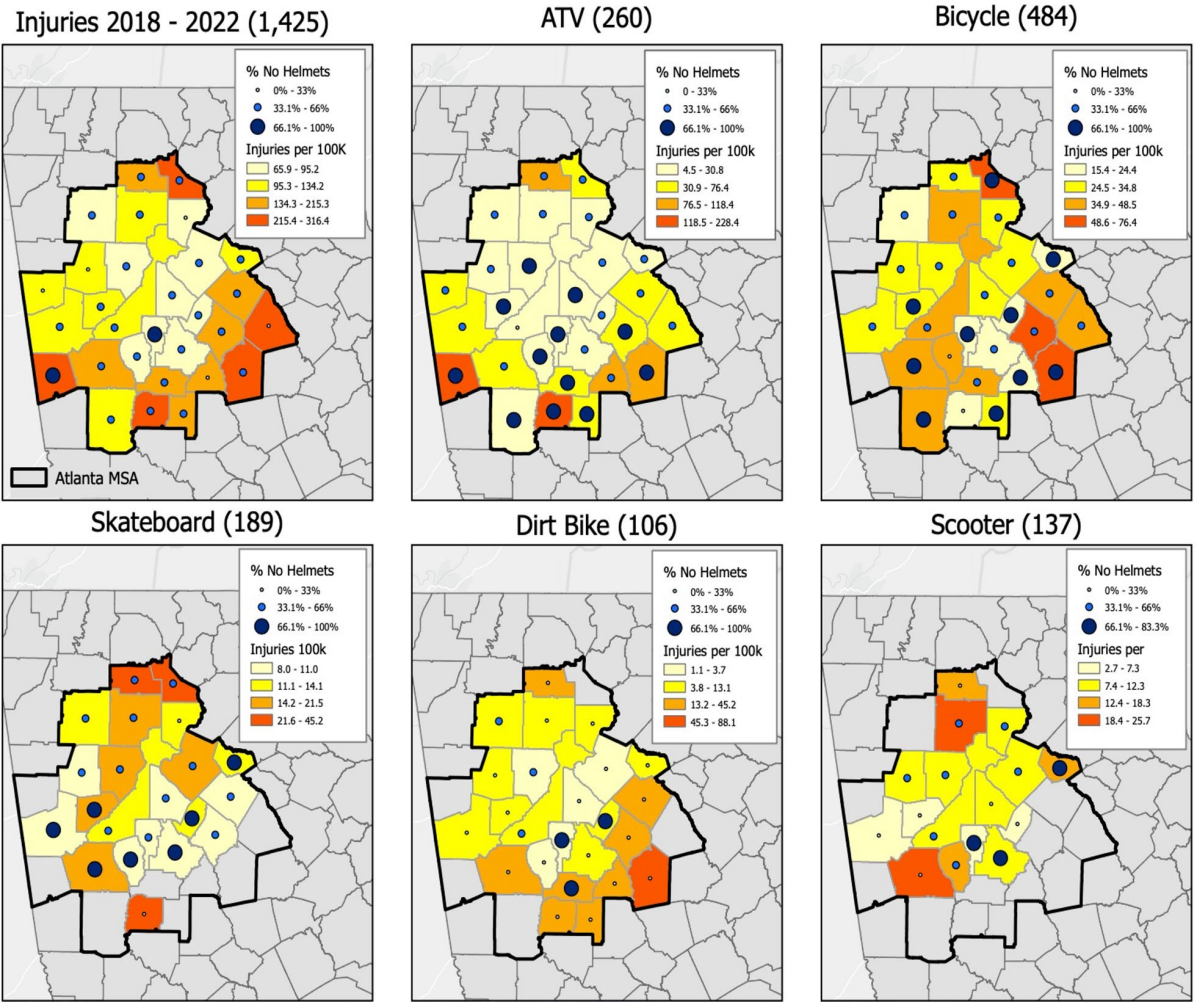
Activities where helmet use is recommended injury rates by no helmet usage. This map displays injury rates for all counties in the Atlanta Metropolitan area from 2018 to 2022, including overall accidents, and is then broken down by the top injury mechanisms

Through GIS mapping, we were able to identify communities at risk for various injury characteristics. By overlapping injury rates for the pediatric population in that community and the percentage of unhelmeted children, we were able to identify high risk communities throughout the years and by top injury mechanisms (Fig. 2).

**Fig. 3:**
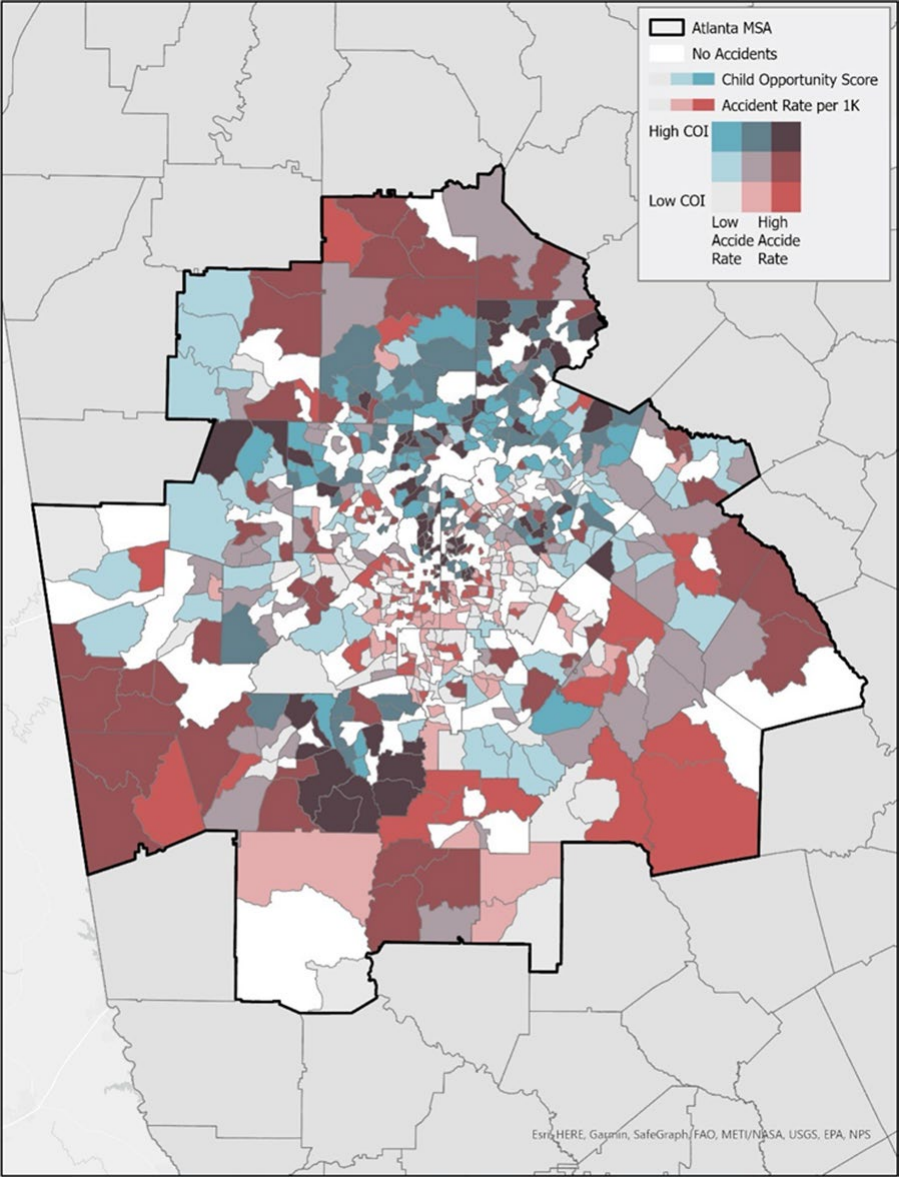
Activities where helmet use is recommended injury rates by childhood opportunity index. This map demonstrates overlapping the injury rates and Childhood Opportunity Index (COI) to identify areas with high injury rates and low COI

Overlapping the injury data with the COI was able to determine communities with lower COI measurement in relation to injury characteristics. Figure 3 demonstrates each COI communities in relation to their injury rate by coloration, with the brighter red color demonstrating communities with lower COI and high accident rates for all activities.

## Discussion

This study uncovered valuable insights regarding the COI and activities where helmet usage is recommended (AWHUR). The majority of children injured during these activities were between 5–9 years old and 10–14 years old for our extracted data from the trauma registry, and there was not a statistical difference in the age groups for the measurements of COI throughout the years. Males were most often involved in the injury for each COI measurement category. This study found similar patterns to those in previous analyses, indicating that black children in our analysis were more likely to be in communities with lower COI measurements [27]. Similar to previous findings, this helps provide context for health inequities in these communities and potential future interventions, as discussed below in conjunction with our GIS mapping.

A significant association was discovered in evaluation of insurance status and the COI. We found that 81.9% of patients injured with very low COI were publicly insured compared to 65.8% of patients injured in high COI areas were privately insured children. This finding follows previous studies evaluating the COI that determined public government insurance was more commonly found for lower COI children and private insurance found more often in higher COI areas [28]. This is concerning as we have seen that children on public insurance are less likely to be helmeted at the time of their injury [29]. We found that children with a lower COI measurement were more often unhelmeted at the time of their injury while children with a higher COI were more often to be helmeted at the time of their injury. This is consistent with previous research that found children with lower socioeconomic factors and being publicly insured are more commonly unhelmeted in their injuries [30–32]. Previous analysis has shown the lack of education contributing to helmet utilization and the increased helmet utilization after educational campaigns [31, 32]. We have also seen that children are less adherent with helmet utilization in communities with socioeconomic disparities [33].

However, when looking at other injury characteristics and the COI, such as head injury, neurosurgery consultation, Injury Severity Score, and Glasgow Coma Score, we did not find statistically significant associations. We did discover that for those who wear a helmet, the odds of having a higher Injury Severity Score was 34% less likely than the odds of those who didn’t wear a helmet. This is consistent with multiple studies and recommendations from the American Academy of Pediatrics on the usage of helmets during these activities [16]. Overall, though, it is important to consider that socioeconomic factors, represented by COI, are associated with less access to resources and care to follow up on the injuries in relation to these activities [34].

Previous studies have seen that certain communities lacked community safety programs impacting helmet usage, in particular due to financial constraints, leading to the importance of GIS mapping and the COI [35]. In addition to the above-mentioned trends on injury characteristics and demographics with the COI, GIS mapping was able to identify specific communities with higher injury rates and lower COI. There was an alarming number of communities that were highlighted with lower COI and higher injuries, as seen in Fig. 3 with the lighter red coloration. Concerningly, we also know that communities such as these, without a pediatric trauma center, have been found in prior studies to under triage pediatric patients leading to poor outcomes and significant disparities across geography with regards to race and ethnicity [9, 10]. Additionally important to consider, there were a few communities with high COI and high injury rates, as we do know that higher COI communities were more often helmeted during the injury event from our analysis. Having this valuable data on injury rates and COI can influence where to most effectively initiate injury prevention campaigns in high-risk communities with less resources [10, 22, 23].

## Limitations

Our study included patient data from the electronic health record and trauma registry, which is dependent on provider documentation. This was a limiting factor, as provider documentation was variable for injury characteristics, such as helmet usage and mechanism of injury clarification. Additionally, for our patient inclusion criteria via ICD-10 codes, provider documentation is a key component to identifying patients who would meet criteria via our trauma registry and without appropriate documentation eligible patients may not be included in our study population. Further, not all patients who presented with injuries during activities where helmet usage is recommended (AWHUR) are included in the trauma registry, leading to patients likely excluded from the study due to our data extraction methods. Another limiting factor was the location, as it would be beneficial to have the location of the injury, in addition to the patient’s home address, used in this study. Additionally, patients not accounted for include those that presented to local emergency departments without referral to our facility, urgent care locations, death prior to the emergency department arrival, or possibly diversion due to saturation of available hospital resources. These potentially unaccounted patients could easily impact a communities’ injury characteristics, as only higher acuity patients are likely transferred to our trauma centers. Additionally, through this bias in higher acuity transfers, we may have higher injury severity rates in communities farther away who can typically manage less severe injuries in their community emergency departments and urgent cares.

## Conclusion

This study highlights the association that children with a lower COI were more likely to be publicly insured with a lower percentage of helmet usage. Alarmingly, concerning patterns are seen in lower COI communities with these childhood injuries in relation to race and ethnicity. We were also able to identify communities with high injury rates and lower helmet usage via GIS mapping. The benefits of identifying the overlapping injury data and COI can identify high risk communities where low resources can contribute to growing injury severity. Potential future interventions in the communities identified via the COI and GIS mapping can help to more effectively allocate resources and interventions. Further analysis is needed into these communities with higher injury rates to determine additional factors related to community resources and their proximity to pediatric trauma care. Population health interventions and campaigns are crucial in our most impacted communities to address safety during these common childhood activities.

## Data Availability

The datasets used and/or analyzed during the current study are available from the corresponding author on reasonable request.

## Abbreviations

TBI: Traumatic brain injury
ED: Emergency department
COI: Childhood opportunity index
AWHUR: Activities when helmet usage is recommended
GIS: Geographical information system
MSA: Metropolitan statistical area
CI: Confidence intervals
OR: Odds ratio
ISS: Injury severity score
